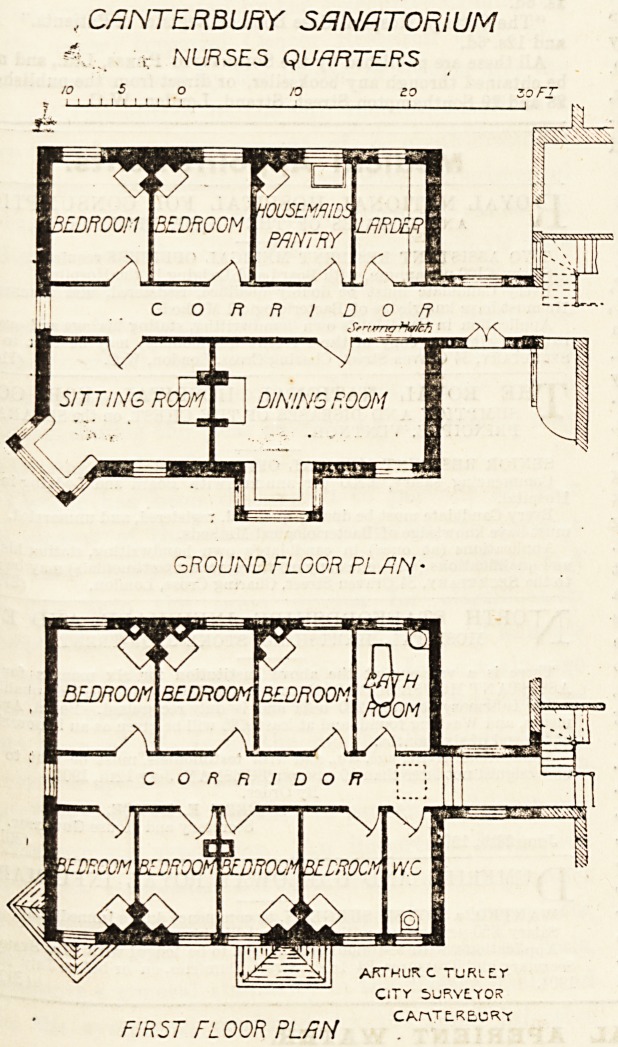# The City Sanatorium, Canterbury

**Published:** 1904-07-09

**Authors:** 


### THE CITY SANATORIUM, CANTERBURY.

A MUCH-NEEDED addition in the form of new quarters for the nurses has lately been put up at the Canterbury Sanatorium. The new building is connected with the administrative part of the old one by a corridor on both floors, and this corridor is cross-ventilated. The new building consists of ground and first floors. The former contains the dining-room, sitting-room, pantry, larder, and two bedrooms; and in the latter space has been found for seven bedrooms and a bathroom. The plan is extremely compact and simple, and provides the required accommodation without waste of space. Indeed, it is so simple, that a detailed description would be wholly unnecessary, as a glance at the accompanying plans would enable anyone to comprehend the design.

The additions permit of each nurse having a separate bedroom, and it has also been found possible to improve the accommodation for domestic servants. The new rooms are comfortably furnished. The sitting-room floor is carpeted; but the other floors are covered with linoleum, than which there is no better material for the purpose. The walls are finished with Duresco of terra-cotta tint. Electric light is used, and the rooms are warmed by open fireplaces.

The architect is Mr. Arthur Turley of Canterbury; and the contractors are Messrs. Harris Bros. The cost is not stated.

## Figures and Tables

**Figure f1:**